# Comparison of 2.4 GHz WiFi FTM- and RSSI-Based Indoor Positioning Methods in Realistic Scenarios

**DOI:** 10.3390/s20164515

**Published:** 2020-08-12

**Authors:** Markus Bullmann, Toni Fetzer, Frank Ebner, Markus Ebner, Frank Deinzer, Marcin Grzegorzek

**Affiliations:** 1 Faculty of Computer Science and Business Information Systems, University of Applied Sciences Würzburg-Schweinfurt, 97070 Würzburg, Germany; toni.fetzer@fhws.de (T.F.); frank.ebner@fhws.de (F.E.); markus.ebner@fhws.de (M.E.); frank.deinzer@fhws.de (F.D.); 2 Institute of Medical Informatics, University of Lübeck, 23547 Lübeck, Germany; grzegorzek@imi.uni-luebeck.de

**Keywords:** fine timing measurement, received signal strength indication, Wi-Fi, position estimation, sensor fusion, smartphone, indoor positioning system, indoor localization, IEEE 802.11-2016

## Abstract

With the addition of the Fine Timing Measurement (FTM) protocol in IEEE 802.11-2016, a promising sensor for smartphone-based indoor positioning systems was introduced. FTM enables a Wi-Fi device to estimate the distance to a second device based on the propagation time of the signal. Recently, FTM has gotten more attention from the scientific community as more compatible devices become available. Due to the claimed robustness and accuracy, FTM is a promising addition to the often used Received Signal Strength Indication (RSSI). In this work, we evaluate FTM on the 2.4 GHz band with 20 MHz channel bandwidth in the context of realistic indoor positioning scenarios. For this purpose, we deploy a least-squares estimation method, a probabilistic positioning approach and a simplistic particle filter implementation. Each method is evaluated using FTM and RSSI separately to show the difference of the techniques. Our results show that, although FTM achieves smaller positioning errors compared to RSSI, its error behavior is similar to RSSI. Furthermore, we demonstrate that an empirically optimized correction value for FTM is required to account for the environment. This correction value can reduce the positioning error significantly.

## 1. Introduction

Many indoor positioning systems (IPS) rely on radio frequency (RF) technologies for estimating a pedestrian’s position inside a building [1]. Probably the most widespread approaches are those based on Received Signal Strength Indication (RSSI) provided by Wi-Fi. Many buildings nowadays offer a good Wi-Fi infrastructure and standard smartphones can be used as the to be located receiver. These two features make Wi-Fi particularly interesting for the application in the consumer market, e.g., navigating inside an airport or shopping mall.

By measuring the RSSI emitted from Wi-Fi access points (AP) at known locations, the receiver, and thus, the pedestrian can be located using the principles of multilateration. Here, at least three APs are needed for two-dimensional positioning and at least four APs are needed for three-dimensional positioning. However, as a simple RSSI-based multilateration is prone to errors in real-world scenarios, causing unacceptable inaccuracies, it is necessary to add more advanced methods to approach the positioning problem in a more accurate and stable way. The two most popular methods are fingerprinting and signal strength prediction [2,3].

Fingerprinting is the process of taking RSSI measurements at known positions distributed throughout the building in a so-called offline or recording phase. The resulting fingerprints are used in the online or positioning phase to obtain the current position by finding the closest match, e.g., by using the nearest neighbor search. Given the current RSSI value, the most likely position is the one which is closest to other similar RSSI fingerprints. This method includes the characteristics of the environment into the prerecorded fingerprints. Consequently, the structure of the building (e.g., walls and furniture) and the positions of the access points need not necessarily be known. Small positioning errors in the lower single-digit meter range can be achieved by using this method. However, the accuracy depends heavily on the density of the fingerprints and whether the smartphone used for the recordings will also be used later for the positioning, as the RSSI characteristics can highly differ between single devices [4]. Recording the fingerprints is a time-consuming and tedious process for large buildings and needs to be redone whenever the environment changes significantly.

In contrast, signal strength prediction methods determine signal strength for arbitrary locations by using an estimation model instead of real measurements. This makes an offline phase unnecessary, but the positions of the access points, as well as additional model parameters, have to be known. Complex scenarios with many non-line-of-sight (NLOS) transmissions require an equally complex signal strength prediction model, taking into account the characteristics of the environment like the different attenuation coefficients of the building’s walls [5,6]. The determination of such parameters is often only possible with a great deal of effort and material knowledge, especially for older buildings [7]. Furthermore, to determine a more or less realistic signal prediction for a location in question, intersection checks of each obstacle within the line-of-sight to the AP have to be calculated. This is computationally costly for large buildings. For this reason, simplified models without intersection checks and precisely determined parameters are often used for the calculation. The positioning errors achieved in this case are often in a reasonable medium single-digit range of meters [8,9].

Since both fingerprinting and signal strength prediction are based on RSSI readings, the accuracy is affected by similar factors. For example, temporary and unpredictable occurrences, such as large crowds are difficult to compensate, as the radio signals are highly attenuated by the human body. Without additional sources like cameras, there can be no indication of where and how many people are in an area. That makes it hard to adapt the above discussed methods.

However, there have been promising alternatives to RSSI for some years now. One of them is time-based distance measurement where on techniques like time of arrival (ToA), time difference of arrival (TDoA) or two way ranging (TWR) are used. As the method names reveal, they are based on the delay the signal took to propagate from the sender to the receiver. Thus, multiplication with the propagation speed of light results in the distance between the two nodes. However, the propagation speed of the signal depends on the propagation medium. It is slower in media with higher relative permittivity compared to air. Consequently, some building elements, like concrete support walls, can add a significant delay to the propagation time, while other materials, like drywall, only add a negligible delay. However, for most indoor environments the signal propagation speed can be assumed to be constant, as the total travel distance in non-air media is usually negligible short compared to the travel distance in air [10]. Because of the direct relation to the propagation time, time-based distance measurements are assumed to be more robust against external interference and environmental changes compared to received power measurements. They also do not depend on AP specific implementation details like reduced transmit strength due to energy-efficiency [11].

Nevertheless, time-based techniques are hardly adapted on a larger scale outside specialized applications or test setups, as they require special hardware. In a smartphone-based system, they are usually not a viable option, as most common devices lack such hardware. This changed with the publication of IEEE 802.11-2016 in 2016, defining the fine timing measurement (FTM) protocol [12]. FTM implements the TWR method for standard conform Wi-Fi devices, measuring the round-trip time (RTT) based on time differences at the sender and receiver. Hence, no synchronized clocks between nodes are needed, which dramatically reduces the complexity of the method and renders a particularly interesting method for smartphone-based positioning.

The aim of this work is to evaluate the applicability of FTM for indoor positioning and if it renders a viable alternative to RSSI. Thus, the reader should get an impression on how FTM can be used inside an IPS and how it behaves in different estimation methods. Therefore, we compare the distance measure obtained by an RSSI-based signal strength prediction model with the distances obtained with FTM. All experiments are done in the 2.4 GHz band. While it is expected that FTM has a smaller error in the 5 GHz band, the results are still interesting for cheap internet of things devices without 5 GHz support.

To make a statement about the performance within an IPS, three different position estimation methods will be introduced and compared, namely multilateration with least-square estimation, a probabilistic positioning approach and a particle filter using a simple transition model. We deliberately do without a full stack IPS to clearly demonstrate the advantages and disadvantages of FTM compared to RSSI.

The paper is structured as follows: in Section 2 we examine papers that are directly related to our work. Section 3 introduces the basic concepts behind RSSI and FTM based distance measurements. The used position estimation methods are presented in Section 4. In Section 5 the particle filter is defined. We show the experimental results, followed by a detailed comparison and discussion of the respective methods and radio technologies, in Section 6. Finally, the work concludes with Section 7.

## 2. Related Work

The FTM protocol was introduced in the IEEE 802.11-2016 standard but it only began recently to get more and more attention in the scientific community. One of the earliest works was presented by Intel [13], where a Kalman Filter and Bayesian Filter were described and compared. They observed that the Bayesian Filter approach with map-matching and smoothing is better suited for FTM due to the non-linear model.

Two years later, Ibrahim et al. [14] gave a comprehensive verification study of the precision and accuracy of FTM with outdoor and indoor measurements in both the 2.4 GHz and 5 GHz frequency band. They conclude that FTM is capable of providing meter-level ranging in open space scenarios, but in the presence of multipath effects the accuracy drops to 5 m indoors. Additionally, Ibrahim et al. published a detailed description on how to setup FTM on Linux with Intel AC 8260 cards, which presumable lead to more publications because off-the-shelf FTM compatible hardware was now available to a broader audience.

Yu et al. [15] developed a complete indoor positioning system based on an FTM ranging model combined with a robust dead reckoning algorithm using an Unscented Kalman filter to fuse the sensors. Their system achieved a positioning error within 2 m. An interesting approach is presented by Choi et al. [16], where unsupervised machine learning techniques are applied to trilateration to adaptively calibrate the range measurements. Their neuronal network indirectly evaluates the accuracy of the range measurements based on the trilateration and iteratively optimizes distance ranging parameters to minimize the error. They reported an average position accuracy of up to 2.397 m with RSSI and 1.547 m with FTM. Dümbgen et al. [17] developed a probabilistic framework which combines multiple sensors based on a conditional random field. Wi-Fi FTM is used as one sensor input besides Bluetooth RSSI, camera images, inertial measurement units and Wi-Fi signal strength. One of the findings in this work is that Wi-Fi FTM measurements are significantly improved by a real-time calibration based on their visual system. They validate their results with two datasets obtained in a large, furnished hall containing glass-windowed lecture rooms with six Wi-Fi FTM access points and reported an accuracy of around 2 m.

Gentner et al. [18] evaluated the FTM accuracy in an antenna measurement chamber and in a typical indoor scenario. They also evaluated the influence of different orientations of the smartphone on the distance estimate and found no dependency. In order to better model the distance estimate error of FTM they used a mixture Gaussian distribution derived from their measurements. This model was then included in the posterior of a particle filter to estimate the position of a moving robot and person. A standard random walk transition model and 3000 particles were used. In their test scenario, they achieved a mean position error of 0.93 m.

In case of NLOS, the distance error of FTM will increase, to mitigate the effect of such faulty measurements on indoor positioning Si et al. [19] developed an NLOS identification algorithm. If such an NLOS measurement is identified, it is discarded. Numerical experiments showed that their algorithm is able to identify NLOS signals with a precision of 83.01% which produced an average positioning error of 0.94 m with their system.

In [20] the author gives a detailed analysis of the error behavior of FTM and describes the underlaying error sources and their contribution to the overall error. Especially the error component depending on the position of the FTM devices has a large effect on the accuracy of the distance estimate. At the same position, this error varies with the frequency. The author showed that the error components in the different channels do not correlate, and thus, allow us to considerably reduce the position-dependent error by a factor $1/\sqrt{6}$. Furthermore, his work gives a comprehensive overview of different positioning methods and introduces a Bayesian grid-based position estimation based on FTM.

Previous work was mainly focused on verifying the ranging accuracy of FTM and integrating it as part of a larger system. The results show that FTM should be well suited for indoor positioning scenarios. However, the application of complex filters and the combination of several sensors makes it hard to reason about the impact of FTM on an indoor positioning system. In addition, most experiments are limited to rather optimistic test setups, where the results are hardly applicable to real-world situations with long and relatively complex walks. For these reasons, the goal of this work is set to directly evaluate the indoor positioning performance with FTM. This is achieved by intentionally using a simple particle filter system without a complex movement model and without integrating other sensors. To evaluate the findings, we compare the FTM results to RSSI based positioning in non-trivial scenarios.

## 3. Wi-Fi Distance Estimate

The nearly ubiquitous Wi-Fi component in modern smartphones is a reasonable choice to be used as a sensor for an indoor positioning system. Wi-Fi is virtually always available, as almost every smartphone comes with an integrated Wi-Fi chip and many buildings have a strong Wi-Fi infrastructure nowadays. Until recently, RSSI was the only data source which could be used with off-the-shelf hardware. Based on signal strength, the distance between the smartphone and the access point can be estimated. As a downside, signal strength heavily depends on the signal propagation, and therefore, on the environment. With the recent advent of FTM in consumer Wi-Fi chips, a feasible alternative to RSSI is now available. Here, the distance between the smartphone and the access point is obtained by measuring the time the signal took to propagate. In the following, RSSI and FTM are discussed in detail.

### 3.1. Received Signal Strength Indication

RSSI is a measure of the received RF power and is obtained by the radio hardware. Its value is usually expressed in dBm and quantified to integer values. For indoor positioning, RSSI can be used to deduce the distance from a smartphone to the access point, because it is virtually always available on common devices [6].

To estimate the distance from RSSI, an appropriate signal strength prediction model needs to be chosen. The log-distance model [21] is commonly used to predict the received signal strength $P_{i}$ from an AP *i* at a given distance $d_{i}$. Formally given as

(1) $$P_{i}=P_{0}-10\gamma \mathrm{lg}\frac{d_{i}}{d_{0}}+X_{\sigma_{i}},$$

where $\mathrm{lg}x=\log_{10}x$ is the common logarithm, $P_{0}$ denotes the measurable signal strength of the AP in dBm at reference distance $d_{0}$; γ is the path loss exponent and must be empirically chosen, based on the given environment. The added zero-mean Gaussian random variable $X_{\sigma_{i}}$ with a standard deviation of $\sigma_{i}\;d\mathrm{Bm}$, models slow fading and random channel noise. Hence, the measured RSSI is assumed to follow a normal distribution $P_{i}∼N\left(P_{i}^{*},\sigma_{i}^{2}\right)$, where $P_{i}^{*}$ is the expected RSSI and $\sigma_{i}^{2}$ is the variance of the measurement.

The log-distance model can be inverted to estimate the distance $d_{i}$ based on the measured RSSI $P_{i}$ and assuming $d_{0}=1m$:

(2) $$\mathrm{lg}d_{i}=\frac{P_{0}-P_{i}}{10\gamma }+\frac{X_{\sigma_{i}}}{10\gamma }$$

(3) $$d_{i}=10^{(P_{0}-P_{i}+X_{\sigma_{i}})/10\gamma }.$$

Since $X_{\sigma_{i}}$ is a Gaussian random variable, the logarithm of $d_{i}$ is normally distributed as well. Consequently, the distance $d_{i}$ follows a log-normal distribution, i.e., $\mathrm{lg}\left(d_{i}\right)∼N\left(d_{i}^{*},\mathrm{lg}\left(\sigma_{i}^{2}\right)\right)$, where $d_{i}^{*}=\mathrm{lg}\left(10\right)\frac{P_{0}-P_{i}}{10\gamma }$ is the expected distance and $\mathrm{lg}\left(\sigma_{i}^{2}\right)={\left(\frac{\mathrm{lg}\left(10\right)\sigma_{i}}{10\gamma }\right)}^{2}$ is the variance of the distance.

In free space, the value of the path loss exponent is γ=2 [21]. In indoor scenarios, γ accounts for the architecture around the AP, thus a constant global factor, which accounts for the whole building, is chosen individually for each AP.

This restricts the log-distance model to a uniform view on the whole environment and does not take the actual propagation path of the signal into account. Therefore, the model does not consider the actual environmental effects or geometry, and thus makes it impossible to differentiate between different types of obstacles or wall materials.

In order to take walls into account, the model must include the power loss of every traversed wall, which results in the wall-attenuation factor model [5]. This requires a detailed map describing the geometry and the semantic properties of the building, e.g., materials, wall thickness, and signal damping factors. Often, the damping factors of walls are unknown or hard to measure. Additionally, walls are often a nonuniform composite of different materials, which makes using lookup tables for damping factors inaccurate. Finally, the computation of the wall-attenuation factor model requires costly intersection tests with the geometry of the environment, which can be intractable to perform on a regular smartphone.

### 3.2. Fine Timing Measurement

As mentioned above, time-based distance measurements are intuitively based on the delay the signal took to travel from the sender to the receiver. An intuitive method to measure the propagation delay of a signal is time of arrival (ToA), where the propagation time of the signal is computed from absolute time values measured at the transmitter and receiver. This method is used famously in satellite navigation, e.g., GPS. While being precise, ToA requires synchronized high precision clocks, which are costly and not practical for indoor positioning.

Two-way ranging eliminates the requirement for synchronized clocks. IEEE 802.11-2016 defines the fine timing measurement protocol, which implements the TWR method for standard conform Wi-Fi devices. The implementation of FTM is optional, still, many manufactures have started to integrate it into their devices. Instead of using absolute time, the time of flight (ToF) is measured based on time differences at the sender and receiver. Synchronized clocks are not required, because successive time measurements are related independently for each device. By definition, the responder, e.g., an AP, is passive while the FTM initiator, e.g., a smartphone, actively requests FTM measurements.

The procedure starts with an initial FTM request frame sent by the initiator, which can be rejected or accepted by the responder. If the responder agrees to the request, it sends an acknowledge frame (ACK). Subsequently, the responder stores the current time $t_{1}$ in memory, which represents the start of the measurement, and sends an FTM frame. The initiator records the time $t_{2}$, as soon as the incoming signal is detected at the antenna. After receiving the FTM frame, the initiator prepares an ACK frame and sends it to the responder. To account for the signal processing delay of the initiator’s hardware, it is necessary to record an additional timestamp $t_{3}$, when the ACK frame is transmitted at the initiator.

When the initiators’ ACK frame is received by the responder at time $t_{4}$ the responder can calculate the total round-trip time of the signal by subtracting $t_{1}$ from $t_{4}$. However, the round-trip time includes the processing time of the initiator, thus, the responder sends $t_{1}$ and $t_{4}$ to the initiator. As the initiator stored its own timestamps $t_{2}$ and $t_{3}$ the propagation time excluding processing delay can be calculated at the initiator. To exclude the processing delay of the initiator, the difference between $t_{2}$ and $t_{3}$ is subtracted from the round-trip time, which results in the time of flight of the signal

(4) $$\mathrm{ToF}=\left(t_{4}-t_{1}\right)-\left(t_{3}-t_{2}\right).$$

Measuring the ToF only once is usually not sufficient. While RF power is relatively simple to measure, obtaining accurate ToF values at a small resolution like nanoseconds requires much more caution, as the measurements are sensitive to noise. Relatively small deviations from the real-time value result in a vast error in the distance estimate, e.g., a measurement error of 10 ns results in a distance error of 3 m. For this reason, the above outlined procedure is repeated multiple times to reduce the impact of noise. In fact, a single FTM measurement or burst instance, consists of many FTM-ACK frame exchanges and the final value ${\mathrm{ToF}}^{*}$ is the average over *N* measurements

(5) $${\mathrm{ToF}}^{*}=\frac{1}{N}\sum_{n=1}^{N}\left(\left(t_{4,n}-t_{1,n}\right)-\left(t_{3,n}-t_{2,n}\right)\right).$$

After calculating the average ToF at the initiator, the FTM measurement process is completed, and the result can be processed by an application layer. With increasing *N*, the impact of noise is reduced, but the delay until the FTM measurement is available for the consuming software increases. Therefore, the actual choice of the value of *N* is a trade-off between precision and measurement delay. Additionally, with larger *N* values, the propagation channel is occupied longer, which increases the likelihood of failing distance measurements, if many FTM initiators try to issue FTM requests [22].

The distance between initiator and responder is half the ToF multiplied by the propagation speed of the signal. Assuming that the signal propagates constantly at the speed of light, the distance between initiator and responder is given with

(6) $$d=\frac{{\mathrm{ToF}}^{*}}{2}\;\cdot \;c+k_{i},$$

where $k_{i}$ is a constant offset in meter. This offset can be used to calibrate hardware-specific delays or systematic errors caused by the hardware [14]. Its value is specified for the given initiator and responder hardware combination.

The accuracy of the distance estimate depends on the accuracy on the recorded timestamps, whose accuracy depends on the used clock and the ability of the hardware to detect the line-of-sight signal, or direct path [23]. In an indoor environment, it is common that a signal will reach the receiver from different paths with different lengths. The prime example is a signal which reaches the receiver via a direct line-of-sight propagation, plus two reflected paths of the same length, e.g., reflected at the walls of a hallway. The direct path is the shortest distance between sender and receiver. The line-of-sight signal will reach the receiving antenna first followed by the reflected signals. However, as both reflected paths have the same length and phase, they constructively interfere at the receiver, resulting in a higher receiving power compared to the direct connection. The difficulty in such multipath scenarios lies in distinguishing the direct path from the reflected paths [14].

Additionally, if the delay of the reflected paths is near the time resolution of the hardware, the multipath components will degrade the precision of the time of arrival estimate. This results in an over-estimate of the propagation time of the signal, and consequently in the distance estimate. The limiting factor is the sampling rate of the receiving hardware, which is defined by the channel bandwidth. Hence, the time resolution is proportional to the inverse of the bandwidth [24].

Consequently, higher bandwidths allow for more precise distance measurements. In IEEE 802.11n, the channel bandwidth is 20 MHz or 40 MHz in the 2.4 GHz range, which results in a sampling rate of one sample every 50 ns or 25 ns, respectively. In case of 80 MHz wide channels in the IEEE 802.11ac 5 GHz range, every 12.5 ns one sample is processed. Assuming that the receiver recognizes the signal at the first sample of the preamble, the smallest possible resolution of the distance estimate is 15 m for 20 MHz bandwidth, 7.5 m for 40 MHz, and 3.74 m for 80 MHz. To allow finer resolution, the receiver uses super-resolution, maximum-likelihood or machine learning methods to allow sub-sample resolution [24,25,26]. However, such implementation details are usually not documented by the hardware manufacture, and therefore, assumed as black box.

## 4. Position Estimation

Multilateration is a geometrical approach to estimate a location based on measured distances between the current unknown position and known reference positions. Given multiple measurements to different reference points, an absolute position in a local coordinate system can be found. In two dimensions, at least three reference points are required to calculate the current position from their distances. The methods from the previous section are used to deduce the current position from the Wi-Fi distance estimates.

With ideal distance measurements to these reference positions, it is straightforward to calculate the current position. However, in the presence of noisy and imperfect measurements, estimating a precise position is a challenging problem. In this section we describe a least-squares estimation method and a probabilistic approach to solve this problem.

### 4.1. Least-Squares Estimation

Given a distance estimate, obtained either with FTM or derived from RSSI, to a Wi-Fi AP as a reference point, the position estimate is constrained by that distance. In two dimensions, each distance estimate $d_{i}$ constrains the position estimate $\hat{\rho }$ to a circle with a radius equal to $d_{i}$, where the center of the circle is the known position $\rho_{i}={\left(x,y\right)}^{T}$ of AP *i*. Formally the measured distance is the Euclidean distance between the known position and the estimate

(7) $$d_{i}=∥\rho_{i}-\hat{\rho }∥.$$

With measurements to several APs, the smartphone position can be found by intersecting the circles defined by (7). Two circles produce two intersection points, assuming that the AP positions are not equal and that an intersection exists. This results in an ambiguity in the estimate as it is unclear which intersection should be selected. A third distance constraint resolves this ambiguity. However, if three or more distances are available, no unique solution can be found that satisfies all the constraints. Additionally, in the presence of noise and inaccurate measurements, an exact analytical solution is impossible.

In this case, an approximation $\hat{\rho }$ can be found by using a least-squares approach, which minimizes the quadratic error between the measured distance and the actual distance at a given point

(8) $$\hat{\rho }=\underset{\rho^{*}}{arg min}\sum_{i}^{}{\left(∥\rho_{i}-\rho^{*}∥-d_{i}\right)}^{2}.$$

This forms a classical non-linear least-squared optimization problem which can be solved with an iterative numerical optimization method like Gauss–Newton algorithm or Levenberg–Marquardt algorithm [27]. The position $\rho^{*}$, which minimizes the error provides an approximate estimate $\hat{\rho }$. In contrast to an analytical solution, this does not result in an ideal point intersection but an area where the error of (8) is acceptably small.

Iterative optimization algorithms require an initial value. Depending on the algorithm, a good initial value is essential for the result. However, the Levenberg–Marquardt algorithm is robust against inaccurate initial values [27]. Therefore, a reasonable choice for the initial value is given by the mean value of all reference points:

(9) $$\rho_{0}^{*}=\frac{1}{N}\sum_{i=1}^{N}\rho_{i}\;,$$

where *N* is the number of reference points [28].

In general, the accuracy of the estimate of (8) depends on the geometry of the setup. In the context of smartphone-based indoor positioning, the multilateration method depends on the position of the APs and the position of the smartphone relatively to each other. To improve the positioning accuracy, it is, therefore, important to consider the actual AP locations and the walkable area, where the system is used.

**Figure 1:**
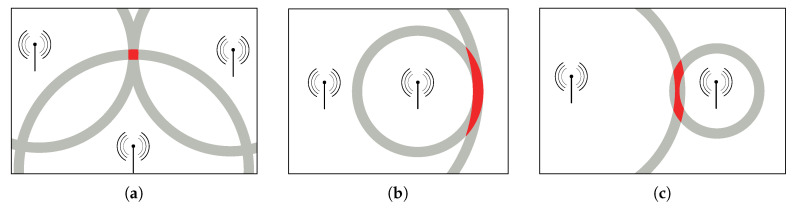
(**a**) Nearly optimal situation with three senders, where the position estimate will be accurate. (**b**) Position accuracy suffers in the vertical direction as both senders lie on a common line. (**c**) With only two senders, ambiguities can occur.

Best positioning results are achieved when the distance circles of the APs intersect in a near orthogonal angle, as seen in Figure 1a. Positioning performance degrades with wider intersection angles, or if the senders and the receiver are placed on a common line, as in Figure 1b. In this scenario a robust horizontal position can be found, due to the narrow shape of the red area. In contrast to the vertical direction, where a larger error is introduced, due to the large height of the area. Ambiguous position estimates caused by non-optimal geometry can occur, as seen in Figure 1c. A more detailed discussion about the placement of the APs is presented in [20].

Usually, non-optimal AP locations need to be chosen due to environmental constrains like building structure. The best geometrical setup for positioning is not necessarily the best setup for signal coverage. The geometrical considerations can be founded on geometric dilution of precision (GDOP), which is a rating of the expected positioning performance based on the sender-receiver geometry [29]. Lower GDOP values indicate better estimation precision due to wider positional separation of the APs as viewed from the smartphone. GDOP allows us to plan the deployment of APs for location estimation before actually installing the APs. While GDOP allows us to rate the given scenario regarding position estimation precision, it only takes the relative geometry of the senders and the receiver into account. RF factors, like signal attenuation or absorption, are not considered. Nevertheless, GDOP still gives a first impression of the theoretical suitability of a geometric arrangement of senders for position estimation and provides an indicator for its further optimizations.

### 4.2. Probabilistic Positioning

The least-squares estimation produces a single point as position estimate by minimizing the quadratic error in (8). The optimization problem is based on the assumption in (7) that the measured distance to an AP is equal to the Euclidean distance between the AP and the current position. This is only true when ideal distance measurements are available. However, sensor data is always noisy and has some measurement error. While the numerical solution allows some measurement inaccuracy, the method cannot model the measurement noise.

A probabilistic framework, on the other side, allows us to describe the estimated position in terms of probability density functions. This allows us to natively incorporate faulty or inaccurate measurements into the position model. With this approach, it is possible to model inaccurate measurements as variances and to quantify them. The likelihood to observe a given position estimate $\hat{\rho }$ is expressed relatively to a known reference point, namely AP *i* at position $\rho_{i}={\left(x,y\right)}^{T}$. Likewise, for every AP a hypothesis about the potential positions, with regard to the given measurement, is made. The combination of all hypotheses results in a single position estimate. However, in contrast to multilateration, $\hat{\rho }$ is not the intersection of circles, but the position with the highest probability given by a density function. While multilateration fails when not enough measurements are available, the probabilistic approach can still produce a hypothesis. As a consequence, it is not always possible to obtain a single point estimate, as there are situations where many positions could be equally likely.

Assuming that FTM measurements follow a Gaussian distribution, the measured distance $d_{i}$ between the sender and AP *i* is described by $N(d_{i},\sigma_{i}^{2})$. $\sigma_{i}^{2}$ is the variance of the measurement. Hence, the probability to observe a distance $d_{i}$ measured to AP *i* at position ρ is given by the density function:

(10) $$p\left(d_{i}|\rho \right)=\frac{1}{\sqrt{2\pi \sigma_{i}^{2}}}\exp\left(-\frac{(d_{i}-∥\rho -\rho_{i}{∥)}^{2}}{2\sigma_{i}^{2}}\right).$$

Visualized on a two-dimensional plane, this produces a ring-shaped density function, where the cross-section of the ring is the well-known Gaussian curve. Multiple distance measurements can be combined by a joint density function if the individual distance measurements to the individual APs are statistically independent. Given the vector $d=(d_{1},\ldots ,d_{i})$, where each component is a distance measurement to an AP *i* at position ρ, the density function of the position estimate is given with:

(11) $$p\left(d|\rho \right)=\prod_{i}p\left(d_{i}|\rho \right)$$

This density gives the likelihood of the position ρ, given the FTM measurements d. The position estimate $\hat{\rho }$ is the most likely position given the measurements

(12) $$\hat{\rho }=\underset{\rho^{*}}{arg max}p\left(d|\rho^{*}\right).$$

The areas, where the individual density functions overlap have higher likelihood to be observed at the given distances. Thus, the geometrical considerations of Section 4.1 still apply to this model.

In the log-distance model (1) the uncertainty is modeled as a zero-mean Gaussian random variable $X_{\sigma_{i}}$ added to the estimated signal strength. Hence, the probability of the position is described in terms of signal strengths instead of distances. Analogous to (10), the likelihood to observe an RSSI value $P_{i}$ from AP *i* at position ρ is given with:

(13) $$p\left(P_{i}|\rho \right)=\frac{1}{\sqrt{2\pi \sigma_{i}^{2}}}\exp\left(-\frac{{\left(P_{i}-P_{i}^{*}\right)}^{2}}{2\sigma_{i}^{2}}\right),$$

where

(14) $$P_{i}^{*}=P_{0}-10\gamma \mathrm{lg}∥\rho -\rho_{i}∥$$

is the expected value given by the log-distance model (1).

Just as with FTM, multiple signal strength values $P=(P_{1},\ldots ,P_{i})$ to several APs are combined to obtain a joint density function, whose mode is the most likely position:

(15) $$p\left(P|\rho \right)=\prod_{i}p\left(P_{i}|\rho \right)$$

(16) $$\hat{\rho }=\underset{\rho^{*}}{arg max}p\left(P|\rho^{*}\right)$$

The probabilistic approach allows us to account for the measurement error by adjusting the value of $\sigma_{i}$. This allows us to dynamically account for measurements of varying accuracy over time.

## 5. Particle Filtering

Indoor positioning is an inherently dynamic process. The primary use case is to estimate the position of a moving pedestrian. Therefore, it is not enough to estimate just a single position, but multiple position estimates at different times are required. This is done by estimating the most likely position at discrete time steps *t*, given the observed values at this time. At *t*, multiple measurements could be available if the measurement rate is higher than the update rate. It is also possible that no measurements are available because of blocked sight to a particular AP.

Moving average filter is a simplistic method to reduce the positioning error. However, this approach introduces a time delay and cannot combine multiple sensors. More advanced filters like Kalman and Bayesian filters use prior knowledge to produce estimates. Measurements observed over time are used and incorporated with a sensor and system model, which allows us to model the measurement noise and system transitions. Kalman filter and its non-linear extensions like unscented Kalman filter assume that the error follows a Gaussian distribution. However, due to the strong variation in human movement and the complexity of different sensor modalities, positioning indoors is often considered as a time-sequential, non-linear, and non-Gaussian model. Bayesian filters update the estimated system state $q_{t}$ recursively with incoming measurements $o_{1:t}$ up to the current time *t* using a set of probabilistic models describing the movement and likelihood. A broad class of methods used to obtain numerical results for this approach are Monte Carlo (MC) methods. By applying the time-sequential hidden Markov process of Bayes filtering, one of the most important MC techniques results: particle filtering.

In the context of indoor positioning, a particle filter computes the posterior distribution $p(q_{t}|o_{1:t})$ describing the pedestrian’s possible whereabouts by using a sample set of *N* independent random variables, $X_{t}^{i}\approx p\left(q_{t}|o_{1:t}\right)$ where i=1,...,N for approximation. Due to importance sampling, a weight $W_{t}^{i}$ is assigned to each sample $X_{t}^{i}$. Thus, ${\left\{W_{1:t}^{i},X_{1:t}^{i}\right\}}_{i=1}^{N}$ is a weighted set of samples, also called particles. A particle is a representation of one possible system state $q_{t}$. Compared to the method described in Section 4.2, this not only allows for a better description of the problem space, but also incorporates a priori knowledge by using a time recursive component.

The filtering equation to calculate the posterior is given by the recursion

(17) $$\begin{array}{cc} \; & p(q_{t}|o_{1:t})\propto \\ \; & \underset{\mathrm{evaluation}}{\underset{︸}{p(o_{t}|q_{t})}}\int \underset{\mathrm{transition}}{\underset{︸}{p(q_{t}|q_{t-1})}}\underset{\mathrm{recursion}}{\underset{︸}{p\left(q_{t-1}|o_{1:t-1}\right)d\;q_{t-1}}} \end{array}\;,$$

where the hidden state $q_{t}$ consist only of a position $\rho_{t}={\left(x_{t},y_{t}\right)}^{T}$ given by

(18) $$q_{t}=\left(\rho_{t}\right).$$

In a more complex filter, the state would consist of multiple quantities. The corresponding observation vector covers all relevant sensor measurements. In this work, only the RSSI value or the distance estimate provided by FTM is exclusively used. For FTM, the observation vector is given with

(19) $$o_{t}^{\mathrm{ftm}}=\left(d_{t,1},\ldots ,d_{t,i}\right)\;,$$

where $d_{t,i}$ are the available distance measurements for AP *i* at time *t* for the current update step. If no distances are available, $d_{t,i}$ is empty. Otherwise, $M_{i}\in N^{+}$ denotes the count of available distances to AP *i* and thus the measurements are defined as

(20) $$d_{t,i}=\left(d_{i,1},\ldots ,d_{i,M_{i}}\right)\;.$$

In case of RSSI the observation vector is analogously defined as

(21) $$o_{t}^{\mathrm{rssi}}=\left(P_{t,1},\ldots ,P_{t,i}\right)\;,$$

with the available RSSI values $P_{t,i}=\left(P_{i,1},\ldots ,P_{i,M_{i}}\right)$ at time *t* for AP *i*.

For the realization of (17) the CONDENSATION particle filter is used [30]. Here, new particles are propagated according to the transition, which models the dynamics of the system. Those particles are then weighted by the evaluation given the sensor measurements. A resampling step is deployed to prevent that only a small number of particles have a significant weight, i.e., to handle the phenomenon of weight degeneracy. These steps are performed based on a predefined discrete update interval or with every new measurement.

As described above, we deliberately do without a full stack IPS in order to clearly demonstrate the advantages and disadvantages of FTM compared to RSSI. We decided to utilize a simple random walk transition model, where the movement of particles from time step t−1 to *t* is provided by drawing from a set of Gaussian distributions. New potential whereabouts $p(q_{t}|q_{t-1})$:

(22) xt=xt−1︷oldpos.+δ︷walked·cos(θ)︷heading,θ∼U(0,2π)yt=yt−1.+δ·sin(θ),δ∼N(μwalk,σwalk)

Note that the uniform distribution in (22) is limited to the interval [0;2π) to avoid oversampling at the pole. Further, the parameters for the Gaussian depend on the chosen update interval, as they describe the to-be-walked distance of the pedestrian. In summary, (22) causes the particles to spread out in a uniformly circular distributed direction within a certain Gaussian distributed distance.

Particle filter approximate the posterior using importance sampling, every particle gets weighted using the probability density of the evaluation in (17). A multitude of different sensor modalities can be incorporated by calculating the product of their respective probabilistic sensor models, which are often assumed to be statistically independent [7]. Note that we also assume statistical independence between the respective AP’s. Within this work we are only interested in a single sensor model at a time, FTM and RSSI, respectively. In case of FTM, the weight of a particle is given by

(23) $$p\left(o_{t}^{\mathrm{ftm}}|q_{t}\right)=\prod_{i}p\left(d_{t,i}|q_{t}\right)=\prod_{i}\prod_{m=1}^{M_{i}}p\left(d_{i,m}|\rho_{t}\right),$$

where $p(d_{i,m}|\rho_{t})$ is the density function of the probability to observe the FTM measurement $d_{i,m}$ at position $\rho_{t}$ from (10). This joint density function allows us to assign a weight to every particle, described by its position $\rho_{t}$, based on the aforementioned probabilistic positioning approach. Likewise, for RSSI the weight of a particle is

(24) $$p\left(o_{t}^{\mathrm{rssi}}|q_{t}\right)=\prod_{i}p\left(P_{t,i}|q_{t}\right)=\prod_{i}\prod_{m=1}^{M_{i}}p\left(P_{i,m}|\rho_{t}\right),$$

where $p(P_{i,m}|\rho_{t})$ is the density function in (13).

Finally, a set of particles ${\left\{W_{t}^{i},X_{t}^{i}\right\}}_{i=1}^{N}$ results after every time interval. As indoor positioning is often seen as a time-sequential problem, we want to provide the best or likeliest position of the pedestrian for the current time step *t*. A fast and intuitive method is to simply select the particle with the highest weight. This is done by finding the likeliest position by calculating the weighted average state $q_{t}^{\mathrm{wa}}$ using

(25) $$q_{t}^{\mathrm{wa}}=\frac{\sum_{i=1}^{N}X_{t}^{i}\;\cdot \;W_{t}^{i}}{\sum_{i=1}^{N}W_{t}^{i}}\;.$$

This does not avoid that the calculated state is somewhere in between the local maxima if the approximated posterior is multimodal. Realistic scenarios are often represented by multimodal densities and therefore it is common that some particles are share the highest weight [31]. In such scenarios, a good way to receive the pedestrian’s position is to recover the probability density function from the sample set itself, by using a non-parametric estimator. As shown in [31], this can be done in a computationally-efficiency manner using an approximation of a kernel density estimator (KDE). Despite reducing the overall variance, it can be observed that such a method does not significantly reduce the error in the general case.

## 6. Experiments

We deployed several experiments to validate the FTM method and to compare it to RSSI. As the FTM hardware support is rather new and experimental, we first evaluate the accuracy of our FTM hardware. This was done in line-of-sight and non-line-of-sight scenarios. In the following, we compare the positioning performance of the methods based on FTM and RSSI, introduced in Section 4.

### 6.1. Hardware Setup

In all our experiments, we used Intel mini PCs running a patched Linux to enable FTM support as access points. Two model generations were used: Intel NUC6CAYS and Intel NUC7CJYSAL. In total, we are using eight APs based on IntelWi-Fi cards. Four of them are based on Intel Dualband-Wireless-AC 8260 cards configured as described by Ibrahim et al. [14]. The remaining four are based on Intel Wireless-AC 9462 modules and run a recent Linux kernel 5.3.7, where the iwlwifi driver and hostapd are already prepared to support FTM. However, the driver still requires small manual changes, as a global Boolean flag needs to be set to activate the FTM related code. In addition, the firmware of the card returns that the chip is not calibrated for FTM. As a consequence, the driver disables FTM responder functionality. Overriding this check, allows us to use the chip as responder and FTM measurements can be presumably performed reliably. At this point, it is not clear to us what the exact purpose of the flag is. While it indicates that the card is not calibrated, the accuracy of the measurements is reliable as will be shown in our experiments. Due to regulatory limitations, both wireless cards can only be configured as access points in the 2.4 GHz frequency band. Furthermore only 20 MHz channel bandwidth is used. Using 802.11n with 40 MHz bandwidth was not possible, due to the coexistence with the university’s existing Wi-Fi infrastructure.

In all our experiments we used a Google Pixel 2 XL and a Google Pixel 3a smartphone, both running Android 9. This is because Google introduced official ranging APIs based on FTM for supported devices with Android 9. Starting with Android 9, Google limited the number of network scans to four every two minutes. This limitation renders the commonly used network scanning API virtually unusable for measuring RSSI values for real-time positioning purposes. However, the new FTM API also provides RSSI values, thus with each measurement, the signal strength and distance to one AP can be simultaneously obtained. More importantly, the new FTM ranging API has no rate limit.

Within the following experiments, FTM measurements to all known access points are queried every 200 ms. This does not guarantee that distance measurements for every access point are available at that frequency. Due to blocked sight or harsh environmental conditions, the measurements can fail. According to our observations, Android issues eight FTM measurements for a single ranging request. The API returns various values, e.g., mean distance, standard deviation, RSSI, number of attempted measurements, as well as the number of successful measurements. It is not documented how Android aggregates the eight measurements, but it is assumed that the arithmetic mean is calculated on the distance values as in (5).

The list of access points is statically stored in the application and known beforehand. With Android 10 it is possible to transfer the AP position dynamically using the location configuration information protocol. This allows for more flexible applications as access points can be added or modified dynamically without updating the client application.

In some cases, we noticed that Android did not provide FTM measurements for intervals of about three and up to five seconds for unknown reasons, although multiple APs were clearly in reach. Due to the rarity of this problem we decided to repeat these faulty experiment runs.

### 6.2. Verification of FTM Performance in LOS Scenario

The first experiment evaluates the indoor precision and accuracy of FTM distance measurements given different hardware configurations. While Ibrahim et al. [14] already verified the precision of the Intel AC 8260 card in great detail, our setup differs from theirs and requires a new evaluation. In contrast to Ibrahim et al. we use smartphones as receivers and an additional card with a newer firmware version as sender. For these reasons we deployed a static distance measurement experimental setup to confirm that the combination of Pixel devices and Intel cards provide reliable values.

**Figure 2:**
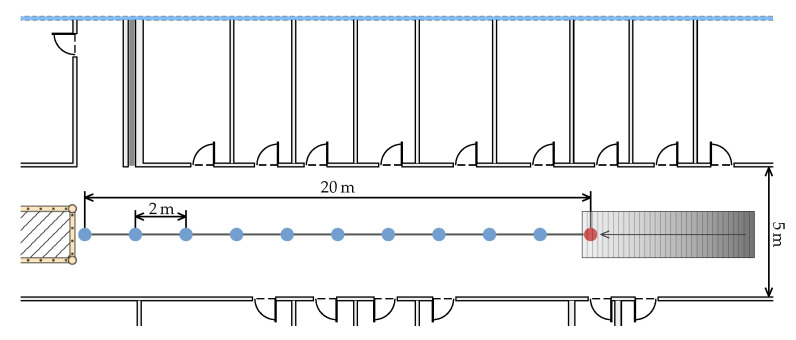
Experimental setup in the main hallway of our university building. Every 2 m 140 Fine Timing Measurement (FTM) measurements were recorded from the smartphone (blue) to the responder (red). The responder is close to a stair.

Our test setup consists of 10 measurement points evenly spaced at a distance of 2 m on a straight line. The closest point to the AP is 2 m away and the furthest 20 m. The setup is shown in Figure 2. At every point, each phone is placed on a stand. Around 140 FTM measurements are recorded, which corresponds to a measure period of 30 s per point. The APs and the phones are placed on an empty cardboard box on a metal stand to allow some distance between the metal and the phone. The box is 12 cm high and the phones laid flat on it. Both the APs and the phones are located at 1.05 m above the floor. Even though recording measurements for 30 s at a single point is not realistic in a dynamic positioning system, it allows us to evaluate the statistical properties of the method.

The whole experiment was deployed in the hallway of our university. Each distance measurement is performed with every hardware combination. We used a Google Pixel 2 XL and a Pixel 3a as FTM initiators and the Intel AC 8260 and 9462 as responders. In total, there are four smartphone-AP combinations.

**Figure 3:**
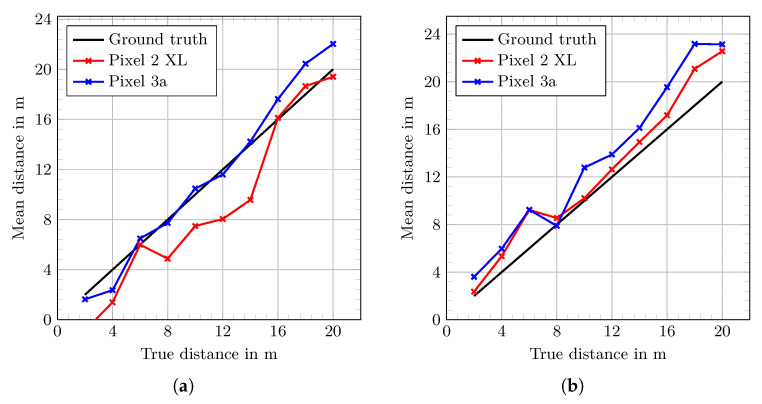
Mean distances using (**a**) the Intel AC 8260 and (**b**) the Intel AC 9462 as responder. The Pixel 2 XL and the Pixel 3a are used as initiators. All measurements were done in the 2.4 GHz band with 20 MHz channel bandwidth.

Figure 3a shows the mean measured distance with respect to the ground truth distance, using the Intel AC 8260 with 20 Mhz channel bandwidth as responder.

Likewise, Figure 3b depicts the mean distances using the Intel AC 9462 with 20 Mhz channel bandwidth as responder. The corresponding values of these figures are shown in Table 1. We compute the mean over the 140 FTM measurements denoted as $\bar{d}$ and its standard deviation. Because $\bar{d}$ can be larger or smaller than the true distance, we used the signed difference between $\bar{d}$ and the true distance as the error metric. However, when it is necessary to quantify the error regardless of its direction the mean absolute error is used, i.e., the mean of all absolute differences between the measurement and ground truth.

Using the Pixel 2 XL as initiator and Intel AC 8260 as responder, the overall error is mostly negative. Contrarily, the Pixel 3a with Intel AC 8260 provides distance estimates close to the ground truth. In case of the Intel AC 9462 as responder both Pixel devices produce similar good values.

**Figure 4:**
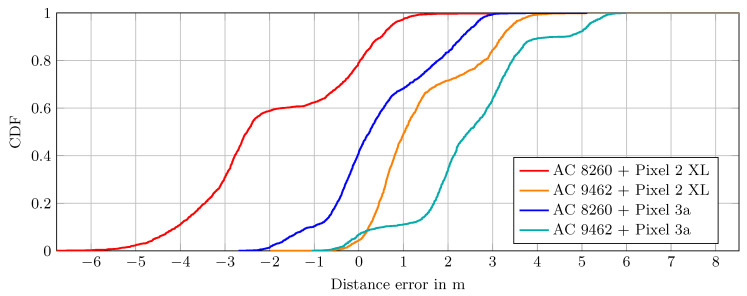
The cumulative distribution function (CDF) of the signed distance error using the Intel AC 8260 and the Intel AC 9462 as responders and the Pixel 2 XL and the Pixel 3a as initiators.

While the mean distance over many measurements is relevant for stationary measure points, in our scenario a pedestrian is moving with the smartphone. Therefore, only one, up to a few measurements, can be observed at a given position. A more expressive visualization for this scenario is given with the cumulative distribution function (CDF) graph in Figure 4, which allows us to reason about the underlying error distribution.

Most striking is the curve of the Intel AC 8260 and Pixel 2 XL combination (red line). Firstly, about 80% of the measurements have a negative error, i.e., underestimate the true distance. Secondly, the curve indicates that the error distribution is a Gaussian mixture distribution with two modes at −3.2 m and 0.088 m, whereas the mode at −3.2 m provides about 60% of the probability mass.

However, using the same card together with the Pixel 3a (blue line), the error is already much smaller and only a small portion is negative. In this case, no constant offset is necessary, as it would not significantly improve the measurements for the Pixel 3a.

In sum, based on the presented results, the Intel AC 9462 seems to provide better results compared to the Intel AC 8260, i.e., most of the errors are positive and up to 5 m.

The results suggest that no single global offset, which accounts for hardware delays, significantly improved the measurements across all device combinations in our tests. However, for each combination of smartphone and access point an individual offset could be used. However, the overall error of the device combinations in a LOS scenario is reasonably small and its distribution is mostly Gaussian-like, which justifies the basic applicability of the technique and the devices for indoor positioning.

### 6.3. Evaluation of FTM Performance in NLOS Scenario

In our experiments, we noticed that at some locations in the building the FTM distance estimates greatly vary compared to other locations. It is likely that environmental factors of the building affect the distance estimation process. Investigating these locations showed that all of them are near special fire doors. These heavy doors are about 12 cm thick, 5 m long, made of metal and presumably grounded. In the case of a fire outbreak, these doors are automatically closed, but normally, they are open and tucked away between walls. Whenever such a fire door is in the line-of-sight between the access point and the smartphone, the ranging error increases significantly. While it is well-known that the environment will affect measurements, especially indoors, it is nevertheless interesting to analyze the underlying cause.

**Figure 5:**
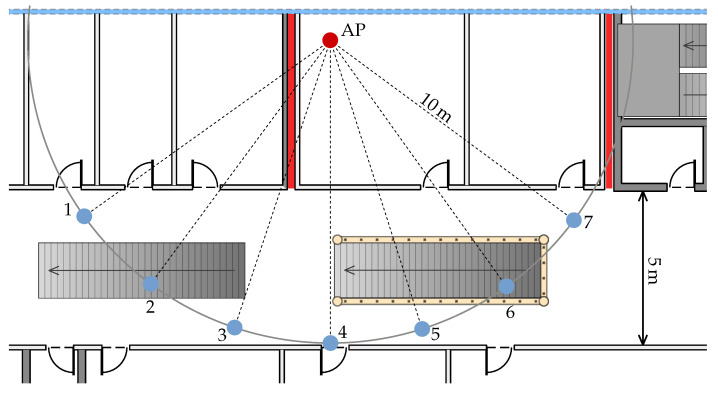
Test setup to evaluate the impact of fire doors (red lines) compared to regular walls (gray). The measurement points are placed on a circle to keep the ground truth distance constant.

To quantify the impact of these fire doors on the measurements we created two test setups using an Intel AC 9462 AP and the Pixel 2 XL. In the first experiment, as shown in Figure 5, we placed seven measurement points onto a circle such that most of these points are located in the main hallway. The radius of the circle is 10 m and measure points 1 to 3 are located in the shadow of the fire door while points 4 to 7 are not. At every point we placed the Pixel 2 XL on a card box on a metal stand at 1.05 m above the floor, just like in the previous experiment in Section 6.2. We recorded FTM distance measurements for 60 s with one measurement every 200 ms. On average, around 255 successful distance measurements were obtained at each point. Note that this number differs from the theoretical possible 300 measurements because some FTM measurements failed.

**Figure 6:**
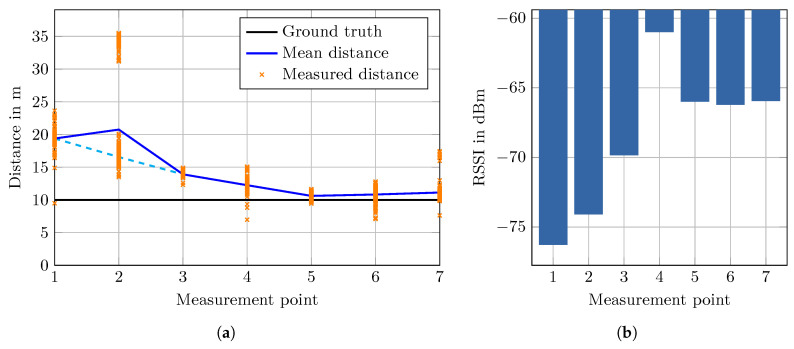
Results for test setup as seen in Figure 5. (**a**) While the true distance (black line) is constant, the mean FTM measured distance (blue line) is not. At point 2, a bimodal distribution of measurements is apparent. If the more significant mode is used the error decreases monotonously (dashed cyan line). (**b**) Corresponding Received Signal Strength Indication (RSSI) values at each measurement point.

The distance measurement results are depicted in Figure 6a. The error in the shadow area is larger compared to the points not shadowed by the fire door. While the measured mean distances at point 1 and 2 are off by around 10 m, the error decreases monotonously for the following points. Points 5 to 7 are not affected by the fire door with a mean error of ≈0.8 m. However, the deviation at point 4, with its signal path quite close to the door, is somewhat larger. The distribution of the distances recorded at point 2 has two modes at 16.55 m and 34.12 m, which are clearly visible in the plot. This bimodal distribution increases the mean distance significantly, if, instead of the mean, the distance at the larger mode ( 16.55 m) is used, then the overall curve is monotonously decreasing (cyan dashed line).

The mean RSSI, as shown in Figure 6b, exhibits the same tendency as the mean distance. At points 1, 2 and 3 the RSSI is decreasing with a minimum at point 4 and stable for the remaining points. This suggests that the RSSI correlates somewhat with the measured distances in this scenario, except at point 4, where the RSSI is stronger than every other point. However, this could be caused by measurement inaccuracy of the smartphone chip and might be a nonrecurring outlier. The RSSI values, compared to the FTM measurements, are stable and have insignificantly small variance in this test.

Notice that point 2, 3, 5 and 6 are located near stairways with massive metal railings. It is expected that the stairways also add measurement noise, however, we still included them deliberately in this test setup as they are nonetheless of real interest because they also appear in the test walks.

**Figure 7:**
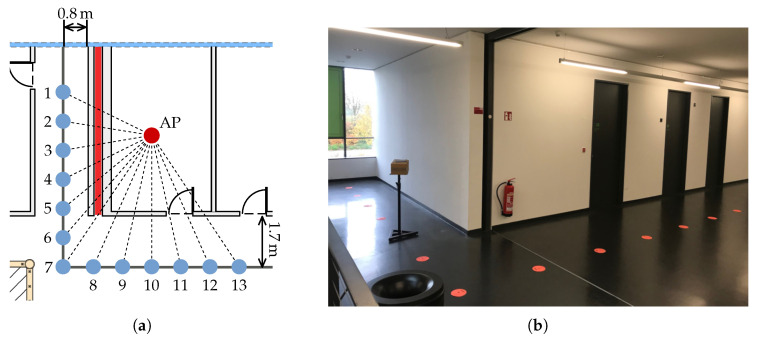
(**a**) Second test setup to evaluate the impact of a fire door (red line) compared to regular walls (gray). (**b**) Photo of the test setup. The fire door is next to the extinguisher tucked away in the wall.

In order to evaluate the effect of the fire door exclusively, we built a second test setup at a corner office located next to a fire door on the same floor. As seen in Figure 7, the 13 measurement points are placed parallel to the wall. Due to structural limitations, it was not possible to keep the distance to the AP constant, like in the aforementioned experiment. The same hardware and placement of the smartphone are used as in the first experiment. The AP is placed in the center of the room on a table. The ground truth was obtained by carefully measuring the right angle distances to walls and taking the line of sight distance from a true to scale map.

**Figure 8:**
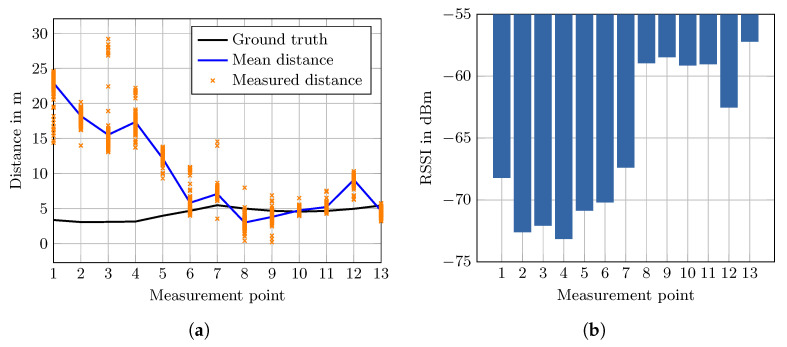
Results for the second setup as seen in Figure 7a. While the ground truth distance only varies slightly (black line) the mean measured distance (blue line) varies greatly depending on the relative position to the fire door.

The results are shown in Figure 8a. Overall, the results shown in Figure 8a are similar to the first experiment but more significant. Again, the error in the shadow area is larger compared to the points not shadowed by the fire door. Measurement point 1 has the largest error with a distance estimate 19.6 m larger than the true distance. Moving towards the end of the shadow area the error decreases nearly monotonously. At point 6, which is still in the shadow area, the error is reasonably small with 1.14 m. The mean absolute error for points 6 to 13 is 1.41 m with a standard deviation of 1.25 m.

Point 12 has a larger error compared to the neighboring points with 4.14 m. This correlates with the measured mean RSSI value at that position (see Figure 8b). As seen in Figure 8a point 12 is located behind the room door. The regular doors in the building are massive and with higher signal attenuation compared to the surrounding drywalls. This could be a plausible cause of the increased error.

However, signal attenuation cannot explain the overall result of the experiment. In both experiments, the true distances to the smartphone are somewhat equal, but the error varies greatly and is larger than the assumed error for LOS. Assuming that the fire door completely blocks any signal, a hypothesis justified by the results is that the signal is received due to multipath propagation. While the measurement points not located in the shadow of the door receive the signal directly, the points in the shadow receive reflected signals with a longer propagation path, and hence, smaller signal strength. This would also explain why point 1 in the second setup has the largest error and the following points have decreasing errors. Without more sophisticated tests, this remains a hypothesis, but the experiments show that these fire doors definitely have a significant effect on the measurements, FTM and RSSI alike, and therefore, on the positioning estimate.

### 6.4. Positioning Environment

**Figure 9:**
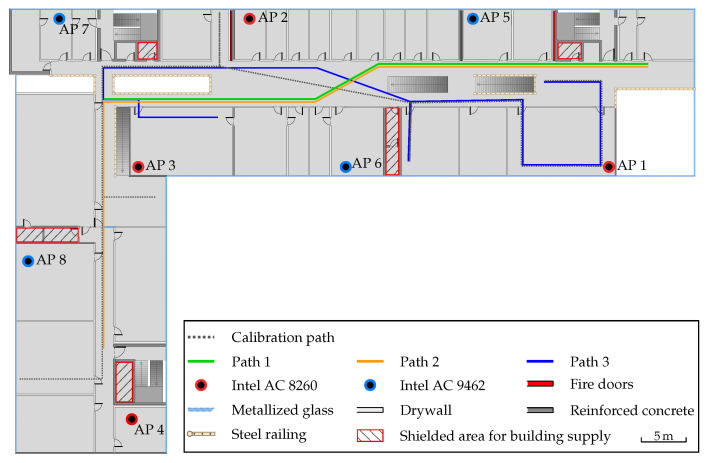
The test environment including the paths of all conducted walks. This is the second of four floors in our university building. Path 1 and 2 started at the right side of the building and are both round trips, that take the same route back. Path 3 started in the same room as where access point (AP) 3 is located. It makes a short detour into a laboratory room and ends after crossing a lecture room at the right end of the building. The calibration path is used as the input to the parameter optimization and was walked in both directions.

All positioning experiments were done on the second of four floors in our university building. It is a modern building with complete external glazing, only interrupted by narrow windows made of metal and plastic. The interior walls are made of drywall except for a few protective or load-bearing elements made of reinforced concrete. In addition to the fire doors already mentioned above, a handful of shielded areas for building supply, like air conditioning, heating system and sanitary installations, are also of particular interest regarding measurement noise. The floor plan including the test setup as well as the walking paths are shown in Figure 9. Path 1 has a length of 124 m and takes 120 s. Path 2 has a length of 180 m and takes 155 s. Path 3 has a length of 112 m and takes 150 s. The calibration walk was chosen to capture the environmental properties and is later used in an optimization step. It is 158 m long and takes 143 s. For each path, 4 different measurement series were recorded. They were carried out consecutively by 2 male testers each using the Pixel 2 XL and 3a at the same day.

A path is indicated by a set of numbered markers fixed to the ground. The ground truth is then measured by manually recording a timestamp while passing a marker. Between two consecutive points, a constant movement speed is assumed. The positioning error is then calculated by comparing the interpolated ground truth position with the current estimation. Thus, the ground truth might not be perfectly accurate, but is inside the tolerance of the error. To record a timestamp, the tester taps on a button on a minimalistic data recording application for Android smartphones. We open-sourced the program code on github.com under GNU General Public License v3.0 [32].

The before-mentioned Intel mini PCs with Intel AC 8260 and 9462 Wi-Fi cards were deployed as APs, because the existing Wi-Fi infrastructure does not support the FTM protocol. While their positions were chosen with positioning in mind, the actual positions mimic the placement of access points for network infrastructure. In contrast to regular stationary access points, which are usually mounted on walls or ceilings, our Intel mini PCs were placed on tables for practical reasons. In our first test runs, we placed the APs near the main hallway or in the center of the room. After some test walks, however, we noticed that moving the APs away from the main hallway actually improves the results significantly. This is also reinforced by a GDOP analysis of the setup, which results in the placement as seen in Figure 9.

### 6.5. Positioning Performance

We compare the positioning performance of the least-squares and probabilistic positioning methods described in Section 4, as well as a simplistic realization of the particle filter as described in Section 5. While it is expected that a filter reduces the positioning error, a less complex method like least-squares estimation allows us to see the characteristics of the sensors better. All three methods were individually executed based on FTM and RSSI measurements. While walking, every 200 ms a FTM and RSSI measurement is issued to each AP (see Section 6.1). Due to the size and environmental factors of the building, it is not possible to obtain a valid measurement for all APs at all times. Therefore, a strategy to deal with missing measurements needs to be considered depending on the used method.

Each distance estimate method has a free parameter: distance offset $k_{i}$ for FTM in (6) and path loss exponent $\gamma_{i}$ for RSSI in (3). Both values account for the environmental effects on the signal caused by the building structure. Hence, their actual values highly affect the performance of the overall system. For each AP we searched for optimized parameters, which reduce the distance error based on the recorded data from the calibration walk. A common set of parameters is used for each path. Table 2 shows the optimized values obtained from the calibration walk, as well as the values for each test walk in comparison. All four walks per path are used as input for the optimization. When deploying an indoor positioning system, the data required for the optimization could be collected in a pre-deployment calibration step. This is similar to fingerprinting approaches, as an additional calibration step has to be conducted beforehand. However, we believe that optimizing the parameters based on a calibration walk, could reduce the setup time compared to classical fingerprinting.

The results of Section 6.2 suggest using individual offsets per smartphone and AP combination. However, we decided to only include the individual APs for the parameter search for practical reasons. When an indoor position system is deployed, we assume that the infrastructure can be controlled to some degree, but not the pedestrian’s smartphone. The smartphone is a much more variable factor as the signals are influenced by the pedestrian, other pedestrians, and the actual holding position of the smartphone. Therefore, including the smartphones in the parameter search might further improve the system but at the cost of higher specialization on the deployed hardware and higher calibration overhead.

The optimized values for the path loss exponent are in the interval [2.5,3.7]. The individual values per AP differ about ±0.2 on average between the three paths. Overall, the three paths are quite similar, as the pedestrian is walking in the main hallway most of the time. Hence, the environmental effects on the signal are also similar per AP and walk. As a consequence, a common average path loss exponent value per AP for every path might be sufficient.

The parameter optimization led to large negative values for FTM, which vary significantly between the paths. Overall, the optimized FTM offsets are in the interval [−10 m,0.65 m]. These offsets compensate for building effects like the fire doors and shielded areas (see Figure 9). The results of the NLOS distance measurements in Section 6.3 indicate that the estimated distances differ from the true distance. Hence, the estimated distances are often larger compared to the true distance. This is corrected by a negative FTM offset. Although not separately evaluated, we assume that the shielded areas have the same error characteristics as the fire doors.

The large negative FTM offset values have a significant downside. Every distance measurement, as reported by the device, which is smaller than the offset, will result in a negative distance after the correction. Negative distances cannot be used for the positioning with the here presented models. The straightforward approach to handle such values is to ignore them for the position estimate. Alternatively, the distances could be clamped to an arbitrary positive value or the original distance measurement without the offset correction could be used. Tests have shown that the actual strategy affects the position error negligibly small, thus we chose to ignore negative distances.

For the least-squares method we use the Levenberg–Marquardt algorithm implementation of the Eigen C++ library to solve (8). The FTM distance values can be directly used in (8). RSSI values are first converted to distance estimates with (3). The FTM and converted RSSI values from one AP are smoothed with a moving average window of size 3. This reduces noise and the impact of outliers but causes a delay in the measurements. Every 500 ms a position is estimated. Assuming that every AP was seen at least once, we could use the averaged value for each AP at every update step to estimate a position. However, we refrain from doing so, to avoid using outdated measurements caused by blocked sight to one AP for a longer period. Only averaged values updated in the most recent time interval are used for the estimation. If there are less than three new values, it is not possible to compute the position with least-squares estimation. In these cases, we use the position estimate from the previous update step for the error computation. This is required to ensure that the method is comparable to the others. While this is not optimal, it shows one downside of the least-squares method without any filters.

For the probabilistic positioning method, we use a grid search to find the mode of the densities (11) and (15). Analogous to the least-squares estimation, a position estimate is computed every 500 ms. Again, one measurement from at least three APs needs to be available in the current time interval. Otherwise, the last position estimate is used for the error calculation. In theory, the probabilistic positioning method has the advantage over least-squares estimation to produce a result in such scenarios. However, if there are less than three distance measurements it is not possible to obtain a single mode but several equally likely positions. This would render the method incomparable to the others. For FTM the distance measurement is used in (10), with $\sigma_{i}^{2}=3.5$ for every AP, which was found empirically. In contrast to least-squares estimation, the RSSI obtained by the hardware is used directly in (13). Again, with an empirically determined $\sigma_{i}^{2}=5.5$ for every AP. The optimized values from Table 2 are used for both methods. If there are multiple measurements available in an update interval for one AP, the values are not averaged. Instead, an additional density $p(d_{i}|\rho )$ or $p(P_{i}|\rho )$ per measurement is added to the joint density.

The particle filter approach is implemented as described in Section 5. We use 5000 particles and compute a position estimate every 500 ms. In contrast to the other two methods, a new position estimate is always obtained, even when there are no new measurements available. The position is estimated with the weighted average over all particles as in (25). The primitive transition model is used without map information. We use $\mu_{\mathrm{walk}}=2.0$ and $\sigma_{\mathrm{walk}}=0.5$ in (22). This allows the particles to spread far enough to capture the jumpy nature of the measurements. The weight of a particle is obtained by the probabilistic positioning method as in (23) and (24) for FTM and RSSI, respectively. Therefore, the reasoning about the probabilistic positioning apply as well.

Table 3 lists the mean position errors and its standard deviation per path, positioning method, smartphone and distance estimation method. Each path was walked four times for each smartphone. The here shown position errors are the mean position error over the four repetitions.

**Figure 10:**
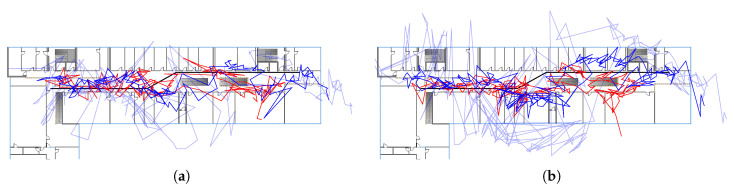
Estimated paths based on RSSI (red), FTM (light blue) and FTM with optimized offsets (blue) using the least-squares method for path 1 with (**a**) Pixel 2 XL and (**b**) Pixel 3a.

In Figure 10 the estimated path for path 1 based on least-squares estimation for RSSI (red), FTM (light blue) and optimized FTM (blue) is shown. In case of RSSI, the position error is similar for the Pixel 2 XL and 3a with 6.4 m and 5.8 m, respectively. For unoptimized FTM, the position error differs significantly between the Pixel 2 XL and 3a with 9.8 m and 12.5 m, respectively. This difference is clearly visible in the estimated path. The Pixel 3a shows much larger jumps and spikes in the position estimate compared to the Pixel 2 XL. The position error is drastically reduced by the optimized values and the estimated path has overall smaller jumps compared to pure FTM. Interestingly, the difference in the position error between the two Pixel devices is also eliminated. Still, the Pixel 3a has visually larger jumps compared to the other device, especially in the middle of the walk.

**Figure 11:**
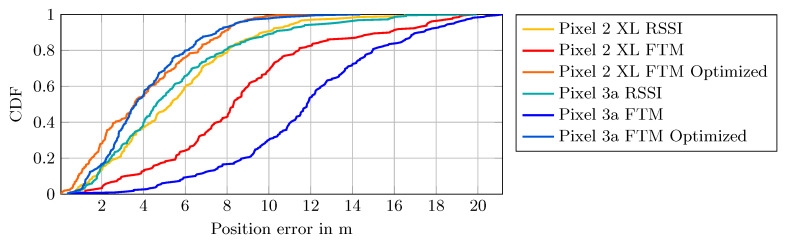
CDF of the positioning error using the least-squares method for path 1. While the RSSI error is similar for both Pixel devices, the FTM error is significantly reduced using the optimized offsets.

The effect of distance correction becomes apparent in the CDF of the position error. Figure 11 shows the CDF of the first path and the least-squares method. The larger position error of the Pixel 3a using the raw FTM measurements is clearly visible in the CDF plot. After the offset correction, both error plots of the Pixel devices are much closer to each other. Additionally, both CDFs are steeper, i.e., the variance in the error is reduced. In contrast, the RSSI position error plots for both devices are already similar without any adjustment.

The performance of the least-squares method for the remaining paths is comparable to path 1. Path 2 is simply an extension to path 1 with an additional detour into the other half of the building. Here, the least-squares method is able to follow the right-angle turn into the additional segment. For path 3, the method is not able to estimate the pedestrian’s position in the rooms. Most estimates are inside the main hallway and not in the rooms, but a tendency towards the rooms is observable. This is visible in the estimated paths, however, the position error does not represent this behavior.

The estimated paths using the probabilistic positioning method are remarkably similar to the least-squares approach. As can be seen in Table 3, the difference in position error is marginal compared to the least-squares estimation. Furthermore, the characteristics of the estimated paths are the same. Both methods produce jumps in the estimated paths and fail to follow the ground truth closely. The similarity to the least-squares estimation is not surprising as both methods implement the same underlying idea. However, as the densities of the probabilistic method are directly used in the particle filter, it can be interpreted as an in-between method. This allows us to see the effect of the particle filtering compared to plain least-squares estimation.

**Figure 12:**
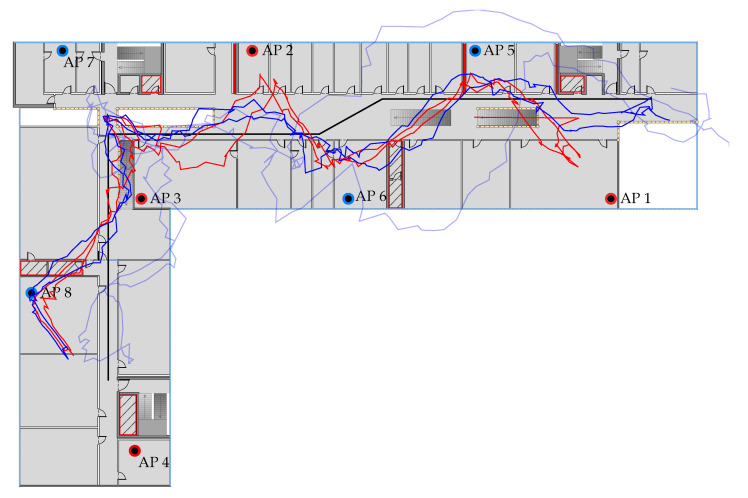
Estimated paths based on RSSI (red), FTM (light blue) and FTM with optimized offsets (blue) using the particle filter method for path 2 with the Pixel 2 XL.

In case of the particle filter, the estimated paths are smoother, and the position error is reduced in comparison to the other two methods. This is expected as the particle filter includes the temporal context. The transition model avoids sudden large changes in the position estimate and the weighted average over the particle set produces a smooth estimate. Figure 12 depicts the estimated path for path 2 using the particle filter method and the Pixel 2 XL. The overall courses of the estimated paths based on RSSI and offset corrected FTM after values are somewhat similar. The position estimate based on FTM has the smaller position error with 3.52 m and $\sigma_{ftm^{\prime }}=2.54\;m$, compared to RSSI with 4.47 m and $\sigma_{rssi}=2.87\;m$.

The estimated path based on RSSI shows a sideways S-shaped curve in the main hallway. Especially at the start and end of the walk the RSSI method fails to follow the ground truth to the start/endpoint but drifts away from the actual position. This is also visible in the left half of the main hallway, where the RSSI path noticeably diverges from the ground truth. Overall, the FTM path has a similar course but is closer to the ground truth at the start/endpoint and in the left half of the main hallway.

In the lower-left of the building, both path estimates are getting “pulled” towards AP 8. The reason for this is, that the smartphone receives almost no measurements from AP 4, as it is shielded by reinforced concrete and a building supply room. Hence, AP 3, AP 7 and AP 8 are the only available reference points in that region. AP 7 is rather far away, and AP 3 is also heavily weakened due to the concrete walls and metal staircase. Furthermore, AP 8 gets distorted most of the time due to the shielded building supply room. Consequently, AP 8 has a large negative offset correction of −10 m (see Table 2). As soon as the pedestrian passes by the supply room the signal is nearly unaltered which results in accurate distance measurements. However, due to the offset correction, these presumably correct distances are significantly shortened, which is apparent in the estimated path.

**Figure 13:**
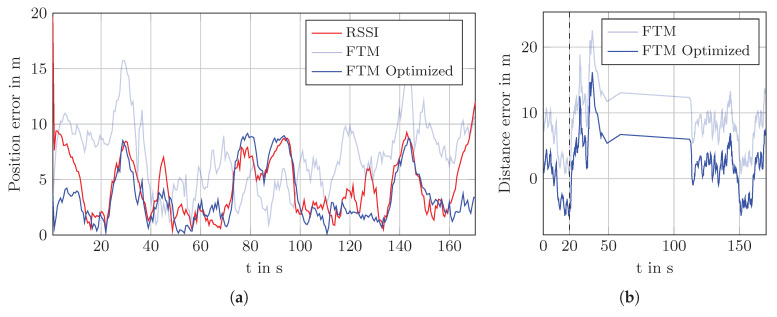
(**a**) Position error for path 2 using the particle filter method and Pixel 2 XL. (**b**) Distance measurement error for AP 5. The distance error is smoothed for better visualization.

This is also visible in the plot of the position error over time in Figure 13a. Between 70 s and 100 s the error of the uncorrected FTM is actually smaller than the corrected one. Outside of that interval, the corrected values produce a much smaller position error. The before mentioned strategy to use the raw FTM measurement when the distance becomes negative after the correction improves this specific situation. However, the difference is only small and only in that situation.

Generally, the positioning performance in the secondary hallway is poor due to the unfortunate placement of the AP 4 and AP 8. However, this circumstance was only noticed after the data was recorded. Better results are expected, when AP 4 is placed to the room in the farthermost left room and AP 8 to the right room across the hallway. Nevertheless, the results make it clear that RSSI and FTM are equally strong affected by the environment and thoughtful placement of the APs is still crucial for the performance of indoor positioning systems.

The same reasoning also applies to the main hallway. AP 5 is placed in a room which is surrounded by two fire doors. As seen in the NLOS experiment in Section 6.3 distance estimates to this AP are much larger if the line of sight between the smartphone and the AP crosses the fire door. Figure 13b shows the distance error for AP 5 with and without offset correction. A positive error indicates that the distance measurement was larger than the true distance and negative value means that the measurement was shorter. The jump in the interval from 50 s to 110 s is due to the absence of measurements to that AP. At 20 s (dashed black line) the error increases abruptly due to the fire door. Likewise, at 150 s the error drops as the pedestrian passes the fire door on his way back. This correlates directly with the drift of the estimated path away from the ground truth path.

The effect of the fire door is also visible in the estimated path based on the unaltered FTM measurements (see Figure 12, light blue line). Here, the path curves around the room, due to the larger measured distances. Note that the transition model of the particle filter randomly moves the particles. Because of that random nature the estimated path can run above or below the room. Again, this is adjusted by the offset correction but, like with AP 8, too much as the estimated path gets “pulled” into the room.

All in all, there are many situations like this, due to the relatively large number of shielded rooms and fire doors in the university building. This is worsened by the small number of APs. Adding more APs to the setup could improve the result, as overall more measurements are likely to be available during the positioning. Alternatively, a more flexible model which incorporates local distortions in the distance measurement should be beneficial.

## 7. Conclusions

In this work, we compared RSSI and FTM-based indoor positioning approaches in non-trivial scenarios in the 2.4 GHz band with 20 MHz channel bandwidth. We deliberately chose simplistic methods like least-squares estimation without advanced filters to expose the characteristics of both radio techniques. In our experiments RSSI, compared to uncalibrated FTM, allows a more stable position estimate which differs moderately between the devices used in this work. Using FTM distance correction values for each AP improves the results for FTM considerably and outperforms RSSI. Better results for FTM are expected with higher channel bandwidths, while RSSI is not influenced by the bandwidth.

The positioning experiments were deployed in a modern building with several fire doors and building supply rooms which present significant interferences to RF signals. Due to the specialized hardware, a relatively small amount of eight access points were deployed into a large building. The eight access points were unintentionally placed at partly unfavorable positions, which made the impact of the building structure more recognizable. These conditions highlighted the importance of the distance correction. It was seen that a one-time calibration of a device is not necessarily required. However, the distance correction poses an important parameter to account for environmental conditions.

Consequently, the actual method to determine the correction values is crucial for the system. In this work, we used the recorded data from a calibration path to optimize the distance correction offset, which reduces the positioning error dramatically. Clearly, this practice might be not sufficient for every real-world application but shows that the positioning performance based on FTM can be improved with a more sophisticated model. For further applications, a full-stack multi-modal particle filter with a real transition model and map information will be applied to more complex scenarios with several floors and rooms. Such an approach would benefit from improved FTM measurements.

## Figures and Tables

**Table 1:** Results for the line-of-sight (LOS) experiment. Mean distance $\bar{d}$, standard deviation σ and the error $\varepsilon =\bar{d}-\mathrm{GT}$ for each ground truth distance (GT). Both Intel cards are used as responders and the Pixel devices as initiators. All values in meters.

	Intel AC 8260						Intel AC 9462					
GT	Pixel 2 XL			Pixel 3a			Pixel 2 XL			Pixel 3a		
	$\bar{d}$	σ	ε	$\bar{d}$	σ	ε	$\bar{d}$	σ	ε	$\bar{d}$	σ	ε
2	−0.857	0.238	−2.857	1.634	0.301	−0.366	2.368	0.383	0.368	3.611	0.415	1.611
4	1.395	0.295	−2.605	2.366	0.351	−1.634	5.333	0.279	1.333	5.968	0.287	1.968
6	5.995	0.526	−0.005	6.496	0.502	0.496	9.226	0.400	3.226	9.241	0.271	3.241
8	4.881	0.327	−3.119	7.725	0.264	−0.275	8.547	0.230	0.547	7.892	0.326	−0.108
10	7.485	0.381	−2.515	10.470	0.482	0.470	10.216	0.322	0.216	12.783	0.241	2.783
12	8.045	0.395	−3.955	11.599	0.329	−0.401	12.635	0.345	0.635	13.884	0.244	1.884
14	9.570	0.790	−4.430	14.230	0.308	0.230	14.941	0.317	0.941	16.116	0.266	2.116
16	16.123	0.728	0.123	17.620	0.406	1.620	17.180	0.452	1.180	19.537	0.327	3.537
18	18.655	0.424	0.655	20.438	0.361	2.438	21.077	0.494	3.077	23.174	0.302	5.174
20	19.404	0.439	−0.596	22.022	0.594	2.022	22.559	0.881	2.559	23.138	0.342	3.138

**Table 2:** The optimized values for path loss exponent and distance offset (in meter) per AP and path. The values obtained from the calibration path are used for the position estimation.

	Path Loss Exponent $\gamma_{i}$								Distance Offset $k_{i}$							
	$\gamma_{1}$	$\gamma_{2}$	$\gamma_{3}$	$\gamma_{4}$	$\gamma_{5}$	$\gamma_{6}$	$\gamma_{7}$	$\gamma_{8}$	$k_{1}$	$k_{2}$	$k_{3}$	$k_{4}$	$k_{5}$	$k_{6}$	$k_{7}$	$k_{8}$
**Calibration** **Path**	2.656	3.313	2.672	3.547	3.203	3.500	3.266	3.594	−0.813	−5.000	−0.156	−2.813	−6.344	−9.750	−4.844	−10.00
**Path 1**	2.625	3.250	2.656	2.922	3.219	3.281	3.109	3.688	−0.781	−1.813	0.219	−7.813	−8.781	−7.344	−6.563	−10.00
**Path 2**	2.708	3.313	2.813	3.396	3.208	3.396	3.146	3.750	−0.375	−1.667	0.583	−3.917	−8.542	−7.667	−5.542	−6.958
**Path 3**	2.531	3.391	2.766	3.250	3.047	3.563	3.313	3.688	0.031	−4.031	0.656	−6.313	−6.250	−9.031	−6.563	−9.844

**Table 3:** The mean positioning error for each path, positioning method (least-squares (LS), Probabilistic Positioning (PP) and Particle Filter (PF)) and smartphone (Pixel 2 XL (P2), Pixel 3a (P3)). The mean position error is listed for RSSI, FTM and FTM with distance correction (FTM′) and its standard deviation σ, respectively. All values in meter.

		Path 1						Path 2						Path 3					
		RSSI	$\sigma_{\mathrm{rssi}}$	FTM	$\sigma_{\mathrm{ftm}}$	FTM′	$\sigma_{{\mathrm{ftm}}^{\prime }}$	RSSI	$\sigma_{\mathrm{rssi}}$	FTM	$\sigma_{\mathrm{ftm}}$	FTM′	$\sigma_{{\mathrm{ftm}}^{\prime }}$	RSSI	$\sigma_{\mathrm{rssi}}$	FTM	$\sigma_{\mathrm{ftm}}$	FTM′	$\sigma_{{\mathrm{ftm}}^{\prime }}$
**LS**	**P2**	6.41	7.47	9.81	7.51	4.78	6.88	5.13	3.26	7.94	3.66	3.77	2.65	5.41	3.34	10.10	5.25	4.23	2.72
	**P3**	5.79	3.82	12.48	4.26	4.41	3.03	5.47	3.28	11.19	4.36	4.62	2.79	5.49	3.11	13.02	5.73	5.25	3.44
**PP**	**P2**	6.38	7.49	9.80	7.48	4.83	6.90	4.98	3.09	7.88	3.73	3.84	2.70	5.27	3.96	9.70	5.23	4.31	2.93
	**P3**	5.40	3.59	11.83	4.46	4.21	3.15	5.19	3.20	10.81	4.25	4.53	2.88	4.91	2.89	12.49	5.72	5.14	3.49
**PF**	**P2**	4.68	2.94	7.66	3.34	3.26	2.10	4.47	2.87	6.62	3.11	3.52	2.54	4.45	2.45	8.40	4.43	3.67	2.00
	**P3**	4.88	3.43	10.91	4.23	3.84	2.70	4.51	2.94	8.90	4.04	3.97	2.38	4.33	2.46	11.48	4.91	4.35	2.76
